# Exploratory factor analysis of PROMIS-29 V1.0, PROMIS Global Health and the RAND SF-36 from chiropractic responders attending care in a practice-based research network

**DOI:** 10.1186/s12955-021-01725-9

**Published:** 2021-03-10

**Authors:** Joel Alcantara, Andrew Whetten, Cameron Zabriskie, Sharad Jones

**Affiliations:** 1 International Chiropractic Pediatric Association, 327 N Middletown Rd, Media, PA 19063 USA; 2 Department of Health and Human Sciences, Southern Cross University, Southern Cross Drive, Bilinga, QLD 4225 Australia; 3 Department of Mathematical Sciences, University of Wisconsin-Milwaukee, 3200 N Cramer St, Milwaukee, WI 53211 USA; 4 Department of Mathematics and Statistics, Utah State University, 3900 Old Main Hill, Logan, UT 84322 USA

**Keywords:** PROMIS, RAND SF-36, Chiropractic, EFA

## Abstract

**Background:**

The SF-36 questionnaire is perhaps the most widely used quality of life instrument in the world today, while the PROMIS instruments continue to gain popularity. Given their continued use in chiropractic research and practice, we examined their latent domain structure using exploratory factor analysis (EFA).

**Methods:**

To uncover latent structures of a large series of measured variables from the PROMIS-29, PROMIS Global Health and RAND SF-36 domains, we defined a factor analysis model represented by the equation $X = \mu + \Lambda F +\epsilon$, where $X = (X_{1} , \ldots ,X_{p} )^{T}$ is the matrix of random vectors corresponding to the domains with a mean of $\mu$ and the covariance matrix $\Sigma ,$ $\Lambda = \{ l_{jk} \}_{pxm}$ denotes the matrix of factor loadings, $F = (F_{1} , \ldots ,F_{m} )^{T}$ denotes the matrix of unobserved latent variables that influence the collection of domains and $\epsilon = (_{1} , \ldots ,_{p} )^{T}$ is the vector of latent error terms. The matrix of item responses X was the only observed quantity with restrictions such that variable scores were uncorrelated and of unit variance with the latent errors being independent with the variance vector $\psi$. The inherited structure of X was expressed simply by $\Sigma = \Lambda \Lambda^{T} + \psi$. Orthogonal and oblique rotations were performed on the $\Lambda$ matrix with this equation to improve clarity of the latent structure. Model parameters $\left( {\mu ,\Lambda ,\psi } \right)$ were optimized using the method of minimum residuals. Each EFA model was constructed with Pearson and Polychoric correlation.

**Results:**

For the PROMIS-29, domains were confirmed to be strongly correlated with Factor 1 (i.e., mental health) or Factor 2 (i.e., physical health). Satisfaction with participation in social roles was highly correlated with a 3rd factor (i.e., social health). For the PROMIS Global Health Scale, a 2-factor EFA confirmed the GPH and GMH domains. For the RAND SF-36, an apparent lack of definable structure was observed except for physical function which had a high correlational relationship with Factor 2. The remaining domains lacked correlation with any factors.

**Conclusion:**

Distinct separation in the latent factors between presumed physical, mental and social health domains were found with the PROMIS instruments but relatively indistinguishable domains in the RAND SF-36. We encourage continued efforts in this area of research to improving patient reported outcomes.

## Introduction

Patient reported outcomes (PROs) are patient self-reported measures such as clinical symptoms, patient satisfaction, and quality of life (QoL) without external input from healthcare providers or others. In this era of evidence-informed practice, PROs have become a necessary if not a mandatory requirement to assess the quality of healthcare systems in the context of Donabedian’s framework of structure (i.e., the ratio of providers to patients), process (i.e., technical process and interpersonal experience of patient care) and outcomes (i.e., safety and effectiveness of care) [1, 2].

The SF-36 (i.e., Short-Form Health Survey and RAND versions) [3, 4] is perhaps the most widely used health-related QoL instrument in the world today. Towards continuing efforts to understanding whether healthcare services are relevant to patients' health status and QoL, the National Institutes of Health funded the creation of the Patient Reported Outcomes Measurement Information System (PROMIS) to create reliable, sensitive and valid universal PRO instruments for diverse populations of children and adult patients [5]. With a T score metric based on Item Response Theory (IRT) and health-focused rather than disease-focused measurement system, the PROMIS measures are quickly being established as standard outcomes for clinical research and practice. With the added characteristics of inclusivity (i.e., applicable to a wide array of patients) and comparability (i.e., measures are comparable across different patients and clinical circumstances), the PROMIS instruments have become attractive outcome measures in chiropractic research and clinical practice [6, 7]. Both the SF-36 and PROMIS instruments have been used in characterizing chiropractic patient populations [6–9]. Given their potential and continued use as PROs in chiropractic, we examined their performance in measuring the QoL of chiropractic patients in a practice-based research network (PBRN). We performed an exploratory factor analysis (EFA) of the RAND SF-36, PROMIS-29 and PROMIS Global Health instruments.

## Methods

The data for our EFA was derived from the baseline dataset examining the QoL of chiropractic patients utilizing the PROMIS-29 V1.0, the PROMIS Global Health Scale and the RAND SF-36. The PROMIS-29 (V1.0) is a collection of short forms measuring the QoL domains of physical functioning, anxiety, depression, fatigue, satisfaction with participation in social roles, sleep disturbance, and pain interference. In addition, pain intensity is measured using a numeric rating system (NRS) (0 = no pain; 10 = worst pain imaginable) [10]. PROMIS Global Health is a 10-item instrument that provides measures of global physical health (GPH) and global mental health (GMH) [11]. Responses to the PROMIS items were converted to their numerical values. The numerical values of the domain specific items were summed to create raw scores. For each PROMIS QoL domain (i.e., anxiety, physical functioning, GPH, GMH), a scoring table was developed to associate the raw scores to a T score metric (i.e., mean of 50 and standard deviation of 10) [12], which was referenced to (and centered upon) the US General population [13]. Larger T scores correspond to higher reported measures of a particular QoL domain.

The PROMIS measures were constructed using IRT models [14] that mathematically generated item characteristic curves (ICCs) that display the probabilities of a response to an items as a function of respondents’ scores on the trait being measured. These response curves for the PROMIS items were estimated on a group of respondents from the first wave of PROMIS data collection [15]. The probability is plotted on the y-axis and scores are plotted on the x-axis. For any score on the x-axis, the response curve with the highest value of y identifies the most probable response. Computer codes are written to identify the most probable responses by score. Once the most probable response at each level of symptom severity or function are obtained for each item on the PROMIS short forms (i.e., Physical Function), the results are “mapped” to a PROMIS T-score continuum (i.e., T-score Maps) that displays the most likely responses for a subset of items. This translates numeric scores into language used by patients to describe their degree of severity or impairment in a given symptom or function. For example, the Physical Function item, “Are you able to do chores such as vacuuming or yard work?” have the following response choices: without any difficulty = 5; with a little difficulty = 4; with some difficulty = 3; with much difficulty = 2; unable to do = 1. The IRT-predicted responses are compared with actual responses from individuals suffering from a number of chronic conditions (i.e., muscular dystrophy, multiple sclerosis, post-polio syndrome, or spinal cord injury). In this way, a person’s score is estimated by identifying which response they chose for each administered item. A person’s level on the trait being measured (i.e., physical function) and an associated standard error can also be estimated using maximum likelihood or Bayesian estimation methods [15]. The degree to which IRT-predicted responses match responses observed in the clinical data utilizes descriptive analyses along with Spearman Correlation Coefficient for each PROMIS item. Rothrock et al. [14], found that the IRT-predicted responses were strongly correlated with participants’ actual responses to their PROMIS short form items (i.e., Fatigue, Anxiety, Depression, and Pain Interference) with r values ranging from 0.762 to 0.950.

The RAND SF-36 is a 36-item questionnaire measuring the QoL domains of physical function, role limitations due to physical health, role limitations due to emotional problems, energy/fatigue, emotional well-being, social functioning, bodily pain and general health. Scoring of the RAND SF-36 is a two-step process [16]. First, pre-coded numeric values were recoded per a scoring key. Each item is scored on a 0 to 100 range so that the lowest and highest possible scores are 0 and 100, respectively. Second, items in the same domain are averaged together to create the 8 QoL domains scores. A higher scoring defines a more favorable health state. We report descriptive statistics (i.e., mean, standard deviation, standard error) of each survey instrument in Table 1.

**Table 1:** Descriptive statistics of quality of life domains of PROMIS and RAND SF-36 instruments

Quality of life domain	Mean (SD)	Mean of SE (SD)
GPH	49.27 (7.04)	4.53 (0.33)
GMH	51.28 (7.50)	3.82 (0.38)
Physical function	51.15 (7.18)	4.92 (2.04)
Anxiety	50.02 (8.79)	4.08 (1.56)
Depression	46.20 (7.32)	4.83 (1.75)
Fatigue	49.36 (8.73)	2.79 9 0.78)
Sleep disturbance	49.01 (7.53)	3.57 (0.45)
Satisfaction in social roles	52.72 (8.08)	3.20 (2.95)
Pain interference	50.95 (8.43)	6.79 (12.09)
Physical function	83.55 (21.06)	N/A
*Role limitations due to physical health	73.11 (36.77)	N/A
*Role limitations due to emotional problems	75.18 (34.25)	N/A
*Energy/fatigue	61.07 (19.10)	N/A
*Emotional well-being	76.95 (14.35)	N/A
*Social functioning	81.99 (23.07)	N/A
*Bodily pain	59.76 (29.21)	N/A
*General health	63.90 (23.14)	N/A

*RAND SF-36; *SD* standard deviation; *SE* standard error; *N/A* not applicable

As described above, responses to the PROMIS items of a particular domain are “mapped” to a PROMIS T-score continuum (i.e., T-score Maps) and have associated with them an underlying uncertainty. This variability in our modelling approach was not considered as we sought to uncover structure pertaining to the raw item responses in each survey instrument. We explored for latent structures by constructing three EFA models corresponding to each of the three survey instruments – PROMIS-29, PROMIS Global Health and RAND SF-36. Consequently, the PROMIS-29 EFA model utilized the raw 29 item responses as the input variables, the PROMIS Global Health EFA model utilized the raw 8 item response giving rise to the GPH and GMH domains and the RAND SF36 model utilized the raw 36 item responses. To uncover implicit or latent structure of a large series of measured variables as described, our EFA models were constructed using the “psych” package [17] in R [18]. Unlike confirmatory factor analysis (CFA), relations between all measured variables and latent factors were considered. Our factor analysis model was represented by Eq. 1 below

1 $$X = \mu + \Lambda F + \epsilon$$

where X $= (X_{1} , \ldots ,X_{p} )^{T}$ is the matrix of random vectors corresponding to the realizations of the responses of the items of a respective questionnaire domain with mean responses for each item denoted by the vector $\mu$ with covariance matrix *Σ*, $\Lambda = \{ l_{jk} \}_{pxm}$ denotes the matrix of factor loadings, $F = (F_{1} , \ldots ,F_{m} )^{T}$ denotes the matrix of unobserved latent variables that influence the collections of domains, $\epsilon = (_{1} , \ldots ,_{p} )^{T}$ is the vector of latent error terms. 

The matrix of item responses, X, for PROMIS29 as an example, was an *m* × 29 matrix where m are rows corresponding to the sample size (i.e., m = 662) and the 29 columns correspond to the 29 items in the raw PROMIS-29 instrument. The matrix X was the only observed quantity with restrictions placed on this model such that the variable scores were uncorrelated and of unit variance with the latent errors being independent with the variance vector. The inherited structure of X can thus be expressed most simply by Eq. 2.

2 $$\Sigma = \Lambda \Lambda^{T} + \psi$$

In order to improve the simplicity and clarity of the latent structure, Varimax rotations were then performed on the $$\epsilon$$$$\Lambda$$ matrix in Eq. 2 [19]. The instrument items were ordinal categorical variables by construction, and as such traditional maximum likelihood estimation methods for EFA were insufficient. Model parameters $$(\mu ,\,\Lambda ,\,\psi )$$ were estimated using the method of minimum residuals [20]. We constructed each EFA model using Pearson and Polychoric correlation using the “psych” package in R [17]. We observed negligible differences in the final EFA results and our final results are reported using Pearson correlations.

Our approach was performed with no a priori hypothesis about the factors. In QoL surveys, it may be conventionally presumed that several of the domains in each instrument have items that dominantly characterize physical or mental health. However, some domains are not explicitly pre-determined to be strictly physical or mental health domains. Subsequent analysis can be performed using CFA, which tests a previously assumed hypothesis of the underlying structures of a PRO instrument. In our EFA analysis, we sought to identify the following:

1. Is there a clear/distinct separation in the latent factors between presumed physical and mental health domains from each PRO instrument? For example, in the 2-latent factor EFA, do the latent factors hypothetically/theoretically represent overall physical and overall mental health?
2. If there is a clear mental and physical health structure—are there domains that have unexpectedly strong or weak correlations with the latent factors?
3. Does EFA uncover clear structural differences in the PROMIS and RAND SF-36 responses?

## Results

Our dataset was derived from a convenience sample of 662 responders (467 females; 195 males). Their average age was 43.88 years (SD = 13.86; range = 18–80 years). The results are reported in three different sections corresponding to each QoL instrument. A 2-factor and 3-factor (when required) EFA was reported and summarized for each QoL instrument. In all cases, orthogonal and oblique rotations were considered with the hypothesis that mental and physical health were correlated along with other potential health domains.

**Fig. 1:**
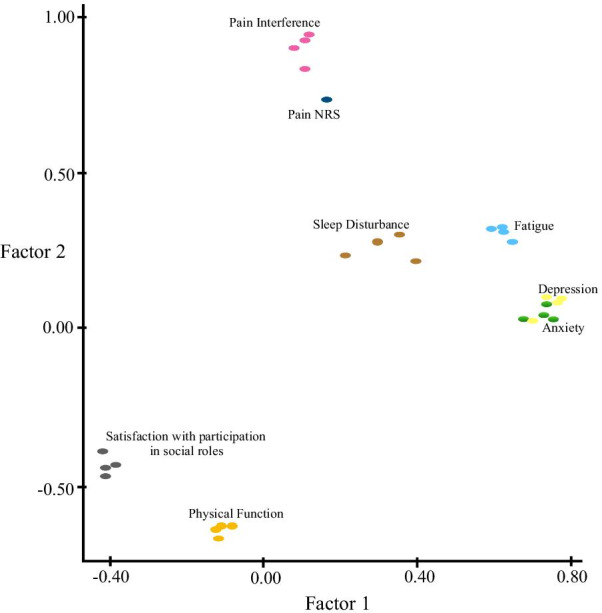
The summary of factor loadings for the PROMIS-29 orthogonal 2-factor model

The PROMIS-29 instrument was examined using both a 2-factor and 3-factor EFA. The summary of factor loadings for the PROMIS-29 orthogonal 2-factor model is provided in Fig. 1. We observed anxiety, depression, and fatigue items were strongly correlated with Factor 1. Pain interference, Pain NRS, and physical function items were strongly correlated with Factor 2. Sleep disturbance had a moderate correlation with Factor 1 while satisfaction with participation in social roles was moderately correlated with both factors. We hypothesize from these results that Factor 1 and the domains anxiety, depression, fatigue uncovers the mental health structure of patients. Similarly, Factor 2 uncovered the physical health structure of our responders. An examination of the oblique factorization was also examined, and the same structures were identified. Table 2 reports the summary of latent factor loading and proportion of variance explained by each factor.

**Table 2:** Summary of latent factor loading and proportion of variance for the PROMIS-29 2-factor EFA

	Factor 1	Factor 2
SS loadings	7.011	1.645
Proportion—variance	0.242	0.238
Cumulative—variance	0.242	0.480

**Fig. 2:**
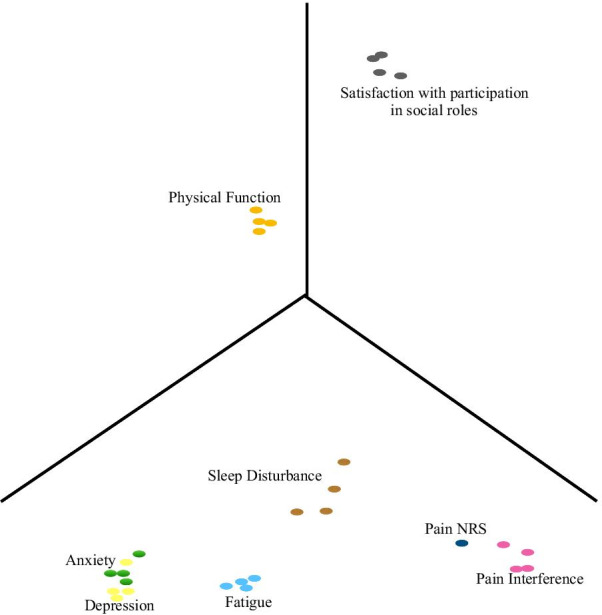
The summary of the factor loadings for the PROMIS29 orthogonal 3-factor model

Since satisfaction with participation in social roles and sleep disturbance items were not highly correlated with either Factor 1 or Factor 2, a 3-factor EFA was constructed with Fig. 2 demonstrating the 3-dimensional (3-D) plot of factor loadings. Since the 3-D plot skews the actual location of the loadings, refer to Table 3 for the loading coordinates based on the orthogonal EFA plot using Varimax rotation.

**Table 3:** Loading coordinates for Orthogonal EFA 3-D plot using Varimax Rotation for the PROMIS-29 instrument

	Factor 1	Factor 2	Factor 3
Anxiety Q1	0.693		
Anxiety Q2	0.753		
Anxiety Q3	0.749		
Anxiety Q4	0.753		
Depression Q1	0.724		
Depression Q2	0.785		
Depression Q3	0.785		− 0.112
Depression Q4	0.765		
Fatigue Q1	0.525	0.273	− 0.283
Fatigue Q2	0.577	0.232	− 0.298
Fatigue Q3	0.552	0.279	− 0.268
Fatigue Q4	0.545	0.261	− 0.297
Pain Interference Q1		0.901	− 0.106
Pain Interference Q2	0.126	0.938	− 0.122
Pain Interference Q3	0.120	0.832	
Pain Interference Q4	0.117	0.929	− 0.156
Pain NRS	0.165	0.720	− 0.139
Physical Function Q1		− 0.622	0.271
Physical Function Q2		− 0.588	0.241
Physical Function Q3		− 0.593	0.213
Physical Function Q4		− 0.590	0.231
Sleep Disturbance Q1	0.281	0.263	− 0.259
Sleep Disturbance Q2	0.321	0.170	− 0.294
Sleep Disturbance Q3	0.266	0.258	− 0.135
Sleep Disturbance Q4	0.200	0.226	
Social Satisfaction Q1	− 0.223	− 0.246	0.799
Social Satisfaction Q2	− 0.192	− 0.287	0.817
Social Satisfaction Q3	− 0.195	− 0.291	0.860
Social Satisfaction Q4	− 0.210	− 0.323	0.830

Anxiety, depression, and fatigue items were confirmed to be strongly correlated with Factor 1 while pain interference, Pain NRS, and physical function items were confirmed to be strongly correlated with Factor 2. Satisfaction with participation in social roles items was found to be highly correlated with Factor 3. This is evidence to suggest that a 3rd latent factor (i.e., social health) improves the uncovered structures in the PROMIS-29 instrument. We found that sleep disturbance items were moderately correlated with all 3 factors. This suggests that sleep disturbance may not exclusively belong to either the physical health, mental health or social health. Rather, the evidence suggests that it is interrelated with all 3 QoL domains.

**Fig. 3:**
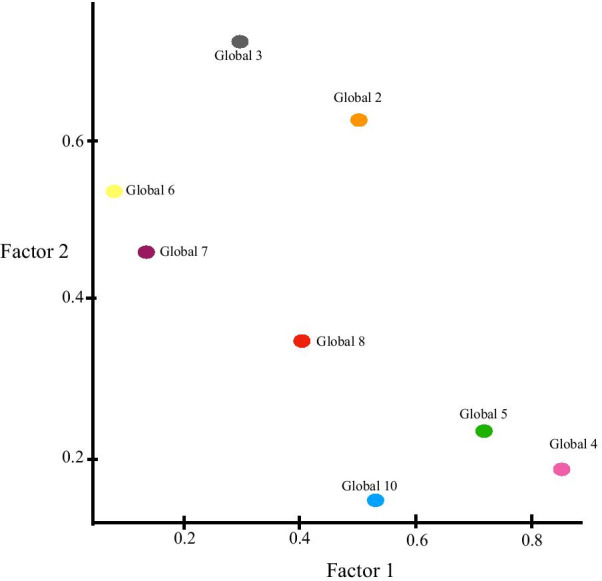
The summary of factor loadings for the PROMIS Global Health orthogonal 2-factor model

**Fig. 4:**
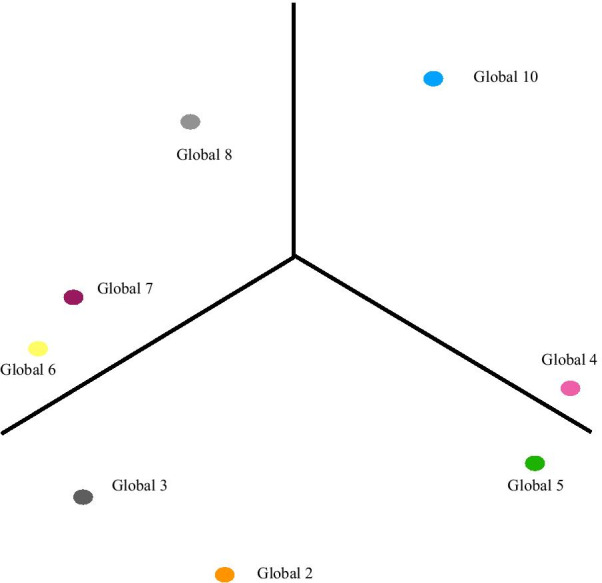
Results of the 3-D orthogonal EFA of PROMIS Global Health

The PROMIS Global Health instrument was examined similarly as the PROMIS-29. The summary of factor loadings for the PROMIS Global Health orthogonal 2-factor model is provided in Fig. 3. Table 4 provides the load and variance summary for this EFA. According to PROMIS [12]*,* items 3, 6, 7, 8 are questions corresponding to GPH. We observed in Fig. 3 that questions 3, 6, 7 are all highly correlated with Factor 2, which suggests that Factor 2 is representative of the latent structure of overall physical health. Items 2, 4, 5, 10 are questions corresponding to GMH. Questions 2, 4, 5 were all highly correlated with Factor 1, suggesting that Factor 1 is representative of the latent structure of overall mental health. Note that item 2 was highly correlated with both Factors 1 and 2 but more correlated with the hypothetical latent physical factor (Factor 2). Item 2 asks responders to rate their overall QoL which provides a sound explanation for its correlation with both Factors. Item 8 was moderately correlated with both Factors. Item 8 asks responders to rate their overall fatigue. As we observed in our PROMIS-29 EFA analysis, we provide evidence that fatigue was not explicitly a physical domain. The summary of factor loadings for the 3-D EFA for PROMIS Global Health is provided in Fig. 4 with Table 5 providing the Orthogonal 3-factor EFA results and Table 6 providing the summary of latent factor loading and proportion of variance explained by each factor. Our findings sufficiently uncovered the 2 structures in the PROMIS Global Health instrument.

**Table 4:** Load and variance summary of the PROMIS Global Health orthogonal 2-factor model

	Factor 1	Factor 2
SS loadings	2.046	1.645
Proportion—variance	0.256	0.206
Cumulative—variance	0.256	0.461

**Table 5:** The Orthogonal 3-factor EFA for the PROMIS Global Health Scale

	Factor 1	Factor 2	Factor 3
Global 2	0.626	0.587	
Global 3	0.365	0.668	
Global 4	0.726	0.128	0.386
Global 5	0.748	0.144	0.215
Global 6		0.541	0.130
Global 7		0.474	0.223
Global 8	0.165	0.365	0.647
Global 10	0.295	0.109	0.652

**Table 6:** Summary of latent factor loading and proportion of variance explained by each factor for Global 3D EFA

	Factor 1	Factor 2	Factor 3
SS loadings	1.732	1.490	1.117
Proportion—variance	0.216	0.186	0.140
Cumulative—variance	0.216	0.403	0.542

**Fig. 5:**
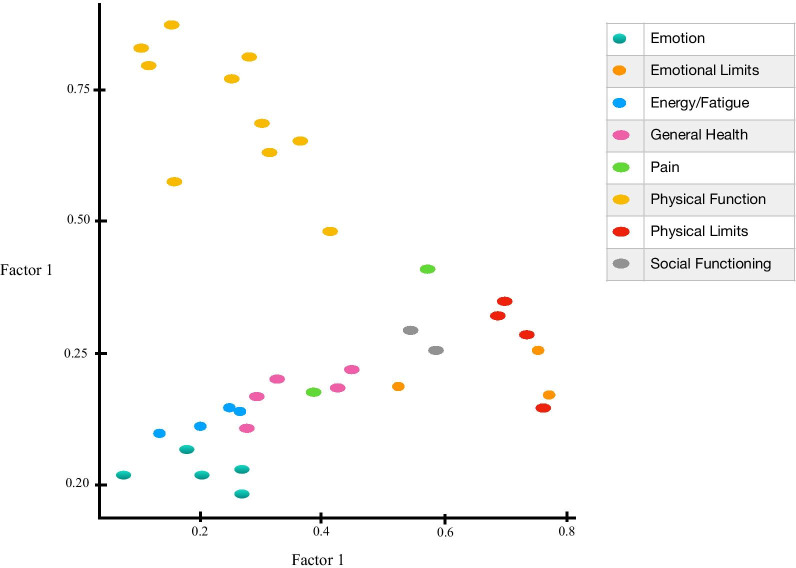
Summary of factor loadings for the RAND SF-36 orthogonal 2-factor model

**Fig. 6:**
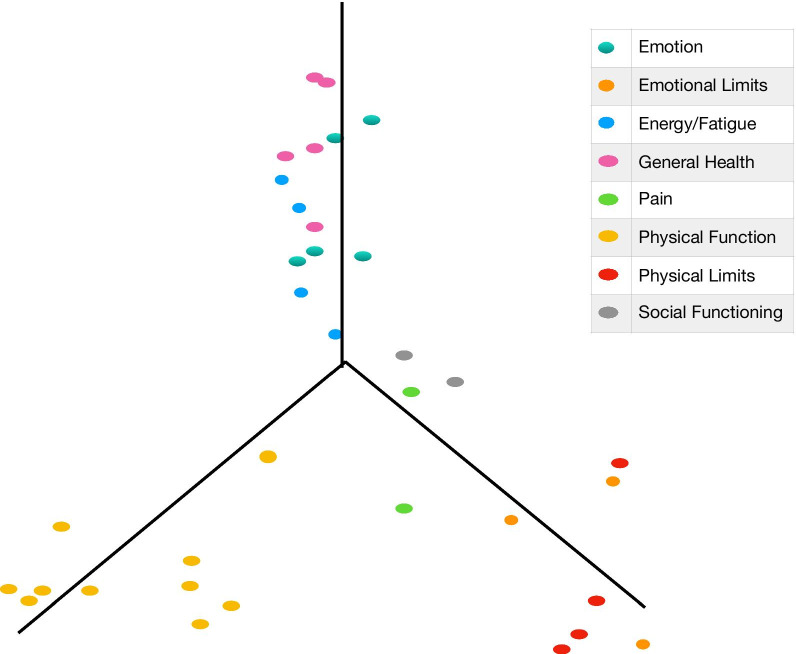
Summary of factor loadings for the RAND SF-36 oblique 3-factor model

The RAND SF-36 questionnaire was examined using both 2-factor and 3-factor EFA. The summary of factor loadings for the RAND SF-36 orthogonal 2-factor model is provided in Fig. 5. Table 7 provides the load and variance summary of this EFA. In Fig. 5, we observed an apparent lack of definable structure. Only the items pertaining to physical function have high correlational relationship with Factor 2 but many of the instrument items (i.e. items corresponding to emotional wellbeing, general health, energy/fatigue) lack correlation with either of the factors. This finding also demonstrated an apparent need for a higher dimensional EFA. The summary of factor loadings for the RAND SF-36 oblique 3-factor model is provided in Fig. 6 and Table 8. Table 9 provides the load and variance summary of this EFA and Table 10 provides the correlations between the oblique latent factors. In Fig. 6, a more informative structure was detected. We observed that Factor 1 was almost exclusively correlated with physical function items. Factor 2 was strongly correlated with emotional wellbeing and role limitations due to physical health as well as moderately correlated with bodily pain. Factor 3 was strongly correlated with emotional wellbeing, general health, and energy/fatigue. Social functioning was not highly correlated with any of the three factors suggesting that a fourth latent factor was likely needed to uncover more of the health structure in the RAND SF-36 instrument. Our exploratory analysis provided compelling evidence that the RAND SF-36 was not designed to assess mental and physical health as distinctly as the PROMIS instruments.

**Table 7:** The load and variance summary of the oblique 2-factor model EFA for the RAND SF-36

	Factor 1	Factor 2
SS loadings	6.299	6.181
Proportion—variance	0.180	0.177
Cumulative—variance	0.180	0.357

**Table 8:** SF36 Oblique 3 Factor EFA loading table

	Factor 1	Factor 2	Factor 3
Q3-Physical Function	0.375	0.166	0.226
Q4-Physical Function	0.595	0.235	
Q5-Physical Function	0.658	0.183	
Q6-Physical Function	0.793		0.143
Q7-Physical Function	0.887	− 0.123	
Q8-Physical Function	0.600	0.122	
Q9-Physical Function	0.818		
Q10-Physical Function	0.952	− 0.138	
Q11-Physical Function	0.924	− 0.140	
Q12-Physical Function	0.605		− 0.109
Q13-Physical Limits		0.845	
Q14-Physical Limits	− 0.173	0.713	0.200
Q15-Physical Limits		0.837	− 0.100
Q16-Physical Limits		0.837	
Q17-Emotional Limits		0.970	− 0.111
Q18-Emotional Limits	− 0.149	0.726	0.183
Q19-Emotional Limits		0.570	
Q23-Energy/Fatigue			0.455
Q27-Energy/Fatigue		− 0.109	0.499
Q29-Energy/Fatigue			0.270
Q31-Energy/Fatigue			0.220
Q24-Emotion		− 0.134	0.279
Q25-Emotion			0.347
Q26-Emotion	− 0.157		0.544
Q28-Emotion			0.328
Q30-Emotion			0.529
Q20-Social Function		0.387	0.328
Q32-Social Function		0.290	0.367
Q21-Pain		0.258	0.206
Q22-Pain	0.216	0.435	0.159
Q1-General Health			0.570
Q33-General Health			0.542
Q34-General Health			0.685
Q35-General Health			0.447
Q36-General Health			0.702

**Table 9:** Load and variance summary for the RAND SF-36 oblique 3-factor model

	Factor 1	Factor 2	Factor 3
SS Loadings	5.673	5.164	3.749
Proportion—Variance	0.162	0.148	0.107
Cumulative—Variance	0.162	0.310	0.417

**Table 10:** The correlations between the oblique latent factors for the RAND SF-36 oblique 3-factor model

	Factor 1	Factor 2	Factor 3
Factor 1	1.000	–	–
Factor 2	− 0.612	1.000	
Factor 3	0.424	− 0.560	1.000

## Discussion

In the scope of assessing the instrument structure of commonly utilized PROs in chiropractic such as the PROMIS-29, PROMIS Global Health and the RAND SF-36, we sought to define “better” QoL instruments. We are of the opinion that QoL instruments should be constructed with a deliberate theoretical structure and that physical and mental health are understood to be interrelated but discernible in the assessment of a patient’s QoL. Even if several domains exist in a QoL instrument, we would argue that an identifiable latent 2-D or 3-D structure should exist to reflect the physical, mental, and potentially social health domains, unless an alternative theoretical structure is proposed.

Based on our analysis, we identified that the PROMIS-29 and PROMIS Global Health instruments have discernible and interpretable physical and mental health structures that was latent in the responses to items within the predetermined domains. When a third latent factor was utilized, a distinct separation was found in our EFA of the PROMIS-29 corresponding to social health (i.e., satisfaction with participation in social roles). Our findings of 3 latent factors with the PROMIS-29 are consistent with the work of Carle et al. [21] Using CFA, Carle et al. [21] tested whether the data collected during the PROMIS Wave 1 field test (2006–2007) corresponded to the theoretical expectations of a physical, mental and social health domain. As others have also found, we observed in our PROMIS-29 EFA of evidence that fatigue and sleep disturbance are not explicitly a physical domain [21–23]. With respect to the PROMIS Global Health Scale, our findings confirmed a 2 domain structure corresponding to GPH and GMH [24]. However, our findings also prompt further discussion regarding an addition of a third domain—general social health to the PROMIS Global Health instrument.

Previous studies have examined the SF-36 independently using EFA and structural equation modeling (SEM) [25, 26]. These papers utilized EFA to inspect the domain latent structure. However, as we performed in our analysis, we proposed that it is best to perform an EFA on the items or survey questions, as this provides evidence of item correlation to latent factors, the proximity of questions to each other within a domain, and inherently, the domain correlation to the latent factors constructed from the questions. In our sample of chiropractic patient responders, all completed the PROMIS-29, the PROMIS Global Health and the RAND SF-36. We performed an EFA on each instrument with the intent of uncovering latent 2-D or 3-D structures within domains and survey items. We provided evidence through this analysis that the items in the PROMIS-29 and PROMIS Global Health have explicit 2-D and 3-D structure and strong groupings of items correlating to the latent factors that is not evident in the RAND SF-36. We also provided evidence of the need to construct survey instruments that examine overall health through general physical, mental, and social health. The RAND SF-36 2-D structure was relatively poor or indistinguishable. When a third latent variable was utilized, we observed a distinct structure, but not necessarily the structure that was hypothesized to exist. We observed that the 3 latent factors relate most strongly to (1) physical functioning, (2) role limitations due to emotional problems and role limitations due to physical health and (3) general health, emotional wellbeing and energy/fatigue. In addition, we found for the possibility of a fourth latent factor that may correspond to a social health domain. Our findings of these 3 latent factors raised inquiries regarding the questions for the RAND SF-36 in terms of wording or choice. Why were physical function and role limitations due to physical health strongly correlated with different latent factors? Why was general health correlated so strongly with the domains that may generally be perceived as mental health domains? Are general health questions generally perceived (as a result of wording) as mental health questions? With no prior theory regarding this unexpected structure in the RAND SF-36 responses, we assert that the PROMIS-29 and PROMIS Global Health are more appropriate instruments for assessing QoL with the ultimate purpose of assessing overall mental, physical and social health.

## Conclusion

Our EFA found distinct separation in the latent factors between presumed physical, mental and social health domains in the PROMIS instruments but with relatively poor or indistinguishable separations in the RAND SF-36. We encourage continued efforts in this area of research to improving the performance of PROs.

## Data Availability

The datasets used and/or analyzed during the current study are available from the corresponding author on reasonable request.

## Abbreviations

RAND: Research and development
SD: Standard deviation
SE: Standard error
SF: Short Form
EFA: Exploratory factor analysis
CFA: Confirmatory factor analysis
GPH: Global physical health
GMH: Global mental health
QoL: Quality of life
2D: 2-Dimensional
3D: 3-Dimensional
N/A: Not applicable
NRS: Numeric rating scale
PRO: Patient reported outcome
SEM: Structural equation modelling
PROMIS: Patient Reported Outcomes Measurement Information System
${\text{X}} = (X_{1} , \ldots ,X_{p} )^{T}$: The matrix of random vectors corresponding to a collection of QoL domains
$\mu$: Mean of the matrix of random vectors
$$\Sigma$$: Covariance matrix
$\Lambda = \{ l_{jk} \}_{pxm}$: The matrix of factor loadings
$F = (F_{1} , \ldots ,F_{m} )^{T}$: The matrix of unobserved latent variables that influence the collections of domains
$\epsilon = (_{1} , \ldots ,_{p} )^{T}$: Is the vector of latent error terms
